# DNA aptamer-functionalized PDA nanoparticles: from colloidal chemistry to biosensor applications

**DOI:** 10.3389/fbioe.2024.1427229

**Published:** 2024-07-09

**Authors:** Ohnmar Zaw, Nang Noon Shean Aye, Jureerut Daduang, Siriporn Proungvitaya, Molin Wongwattanakul, Nipaporn Ngernyuang, Sakda Daduang, Nikorn Shinsuphan, Rungrueang Phatthanakun, Nichada Jearanaikoon, Pornsuda Maraming

**Affiliations:** ^1^ Centre for Research and Development of Medical Diagnostic Laboratories, Faculty of Associated Medical Sciences, Khon Kaen University, Khon Kaen, Thailand; ^2^ Center for Innovation and Standard for Medical Technology and Physical Therapy, Faculty of Associated Medical Sciences, Khon Kaen University, Khon Kaen, Thailand; ^3^ Chulabhorn International College of Medicine, Thammasat University, Pathum Thani, Thailand; ^4^ Thammasat University Research Unit in Biomedical Science, Thammasat University, Pathum Thani, Thailand; ^5^ Division of Pharmacognosy and Toxicology, Faculty of Pharmaceutical Sciences, Khon Kaen University, Khon Kaen, Thailand; ^6^ Medical Instrument Subsection, Maintenance Section, Faculty of Medicine, Chiang Mai University, Chiang Mai, Thailand; ^7^ Synchrotron Light Research Institute (Public Organization), Nakhon Ratchasima, Thailand

**Keywords:** polydopamine nanoparticles, surface modification, DNA aptamer, electrochemical biosensor, molecular dynamics simulation, glycated albumin, polymeric nanoparticles

## Abstract

Polydopamine nanoparticles (PDA NPs) are widely utilized in the field of biomedical science for surface functionalization because of their unique characteristics, such as simple and low-cost preparation methods, good adhesive properties, and ability to incorporate amine and oxygen-rich chemical groups. However, challenges in the application of PDA NPs as surface coatings on electrode surfaces and in conjugation with biomolecules for electrochemical sensors still exist. In this work, we aimed to develop an electrochemical interface based on PDA NPs conjugated with a DNA aptamer for the detection of glycated albumin (GA) and to study DNA aptamers on the surfaces of PDA NPs to understand the aptamer-PDA surface interactions using molecular dynamics (MD) simulation. PDA NPs were synthesized by the oxidation of dopamine in Tris buffer at pH 10.5, conjugated with DNA aptamers specific to GA at different concentrations (0.05, 0.5, and 5 μM), and deposited on screen-printed carbon electrodes (SPCEs). The charge transfer resistance of the PDA NP-coated SPCEs decreased, indicating that the PDA NP composite is a conductive bioorganic material. Transmission electron microscopy (TEM) and scanning electron microscopy (SEM) confirmed that the PDA NPs were spherical, and dynamic light scattering (DLS), Fourier transform infrared spectroscopy (FTIR), and Raman spectroscopy data indicated the successful conjugation of the aptamers on the PDA NPs. The as-prepared electrochemical interface was employed for the detection of GA. The detection limit was 0.17 μg/mL. For MD simulation, anti-GA aptamer through the 5′terminal end in a single-stranded DNA-aptamer structure and NH_2_ linker showed a stable structure with its axis perpendicular to the PDA surface. These findings provide insights into improved biosensor design and have demonstrated the potential for employing electrochemical PDA NP interfaces in point-of-care applications.

## 1 Introduction

Modern concepts in materials science, surface coatings, and surface modifications play important roles in many biomedical applications because surface characteristics and functionalities, such as compatibility with biological systems, biointegration, stability, antifouling performance, and microbial and biofilm activities, are among the most important properties related to nature and coating properties (Hu et al., 2023). The more relevant surface properties are also related to chemical structure (hydrophilicity and the existence of functional groups that could be responsible for the characteristic chemical reactions in biological systems, physical and mechanical properties, morphology, and surface topographical assets) (Ferreira et al., 2015). The development of biosensors and biomedical devices necessitates the use of surfaces that are highly biocompatible and have abundant reactive functional groups. The currently available techniques for the surface modification of materials, including chemical reactions, hydrolysis, layer-by-layer deposition, microwave irradiation, plasma treatment, and nanoparticle growth, are typically time-consuming and complicated processes, and they are not applicable to all surfaces (Liu et al., 2014; Ansari et al., 2023). Therefore, research and innovation efforts in this field aim to discover a coating approach that is effective, simple, and universal, a challenge that remains unresolved.

In recent years, polymeric nanoparticles have garnered significant attention due to the unique properties conferred by their small size, which ranges from 1 to 1000 nm, and can be modified with active ingredients on polymeric surfaces or entrapped within the polymeric matrix (Zielińska et al., 2020). Polymer-based nanoparticles are constructed from various materials, such as chitosan, cellulose, starch, polydopamine (PDA), and alginate (Kumar et al., 2012; Han et al., 2018; Aguilar-Ferrer et al., 2022). The selection of polymer type depends on multiple design options and application requirements. Many factors, such as the nanoparticle size, the properties of the active compounds (aqueous solubility and stability, etc.) to be encapsulated in the polymer or conjugated onto the surface, surface characteristics and functionality, biocompatibility, toxicity, and release profile, are important (Kumar et al., 2012).

Among the possible alternative coating materials, PDA is a nature-inspired biopolymer with a dark brown to black color that is produced from the oxidative polymerization of dopamine or other catecholamines (Ball, 2018). PDA was first reported by the Messersmith group in 2007, who were inspired by the adhesive proteins found in mussels (Liu et al., 2014; Hauser et al., 2020). PDA exhibits a range of properties that make it an attractive material for various applications. It has strong adhesion properties, enabling it to form thin, uniform coatings on a vast variety of materials, including metals, nanoparticles, nanotubes, hydrogels, and electrodes (Palladino et al., 2019; Niyonshuti et al., 2020; Hemmatpour et al., 2023; Yu et al., 2023). It is also biocompatible, making it suitable for applications in medicine and biology. Moreover, PDA has catechol, amine, and imine functional groups, which can serve as the starting points for covalent modification with desired molecules (Chen et al., 2022). The method for preparing PDA through oxidative polymerization on surfaces is an easy procedure but presents challenges in controlling both the spatial localization and surface morphology of the deposited PDA thin film (Loget et al., 2013). Additionally, the formation of aggregates on the film surface can be a common issue during the PDA self-deposition process, which results in reduced PDA film quality and application difficulties (Wang et al., 2023). This major drawback led to the production of PDA in the form of nanoparticles with colloidal stabilization.

Polydopamine nanoparticles (PDA NPs) have been applied for the development of biosensor platforms. The self-adhesive properties of PDA are used to attach nanomaterials onto the electrode’s surface and add chemical groups that can be utilized to immobilize recognizing species (e.g., antigens, antibodies, nucleic acids, aptamers, enzymes, or cells as receptors) for biosensor development (Rocha et al., 2023). The surface modification of PDA NPs with COOH-PEG-Silane provides carboxyl groups for subsequent antibody conjugation (Liu et al., 2023). The PDA NP-antibody complexes were used for colorimetric lateral flow immunoassay (LFIA) methods for the detection of COVID-19 (Liu et al., 2023), *Salmonella typhimurium* (Yang et al., 2023), and Zearalenone (Xu et al., 2019). PDA NPs can absorb spectrum covering the entire UV-visible and near-infrared range, making them extremely efficient fluorescence quenchers. When fluorescent dyes are attached to bioreceptors, the complexes lose their fluorescence upon interacting with the PDA NPs. However, the fluorescence is recovered in a dose-dependent manner when the bioreceptor-bound nanospheres are exposed to the target (Qiang et al., 2014; Ball, 2018).

In electrochemical biosensors, surface modification of electrodes is important for enhancing the selectivity, sensitivity, and other characteristics of the electrodes (Raeisi-Kheirabadi et al., 2022). Nanoparticles have been implemented for surface modification of electrodes. They can immobilize biomolecules and amplify signals due to their large surface area. The use of PDA NPs in sensors represents a growing area of interest. However, most studies have incorporated PDA films onto nanoparticles at the interface of electrochemical sensors (Rocha et al., 2023). Therefore, the use of PDA NPs directly on electrode surfaces conjugated with biomolecules is still a challenge.

In earlier reports, a computational approach was used to study the structural and dynamic characteristics of DNA aptamer secondary structures via molecular dynamics (MD) simulations (Mei et al., 2018; Araujo-Rocha et al., 2021). Studies have used MD simulations to predict crucial information about the DNA aptamer binding site location and orientation as well as the functionalized surface, whose conformational changes may influence the resulting biosensor’s properties. One material substrate that has gained popularity is the PDA surface, which can be used to enhance the structural and surface characteristics of biosensors while also increasing their efficiency (Chen and Buehler, 2018; Li et al., 2021; Zhang et al., 2021; Xin et al., 2024).

**SCHEME 1:**
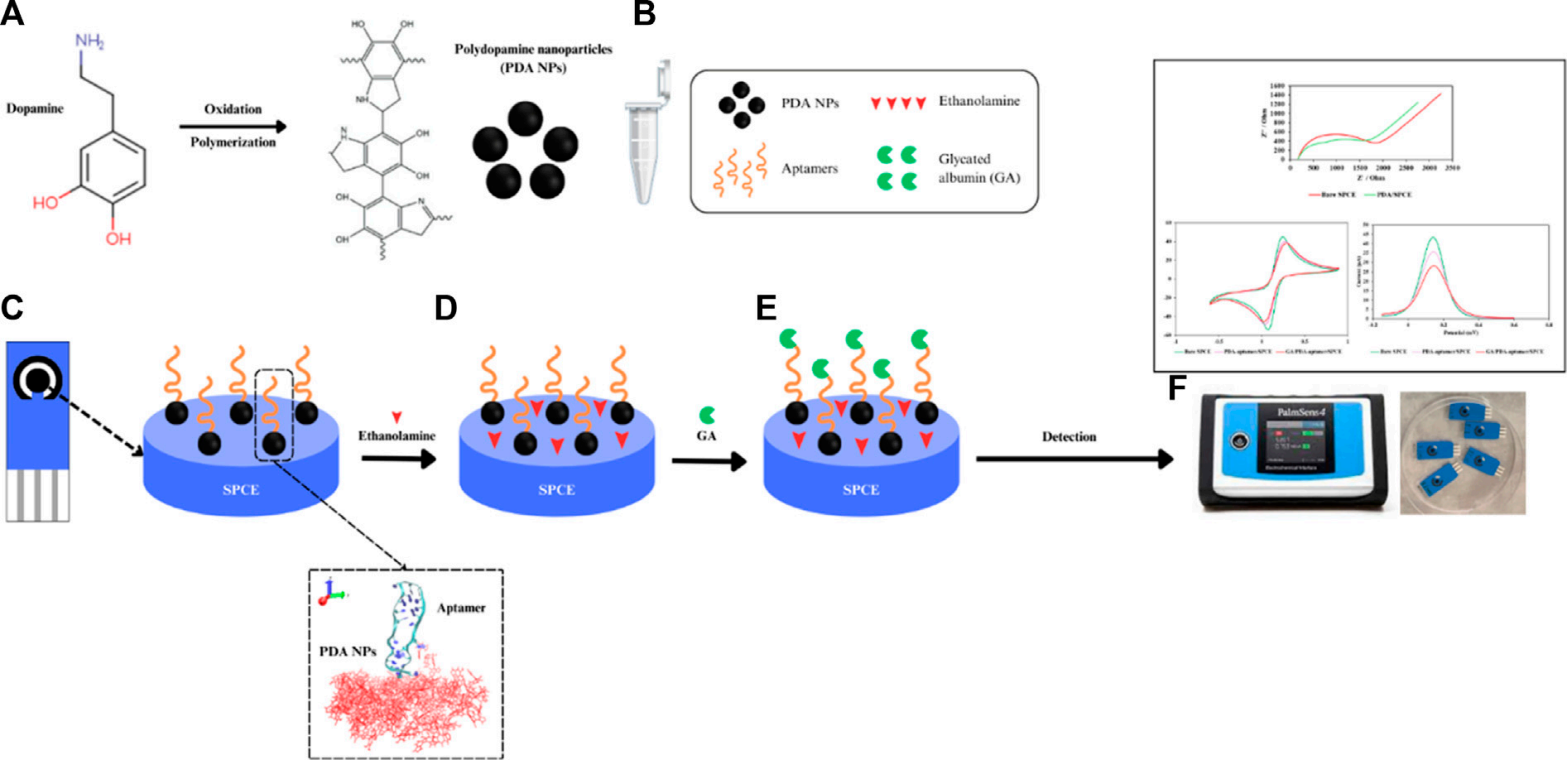
Diagram of an electrochemical aptasensor based on DNA aptamer-functionalized PDA NPs for the detection of GA. **(A)** Synthesis of PDA NPs, **(B)** conjugation of the NH_2_ aptamer and PDA NPs, **(C)** coating of the PDA NP-functionalized aptamer onto the SPCE, **(D)** blocking with ethanolamine, **(E)** incubation with GA and **(F)** voltammetry measurements.

This research delves into the synthesis of PDA NPs and their conjugation with different concentrations of aptamers, aiming to elucidate the intricate chemistry that governs the functionalization process. Characterization of PDA NPs and aptamer-functionalized PDA NPs through techniques such as transmission electron microscopy (TEM), scanning electron microscopy (SEM), dynamic light scattering (DLS), Fourier transform infrared spectroscopy (FTIR), and Raman spectroscopy not only confirmed successful synthesis but also provided insight into the physicochemical properties of the conjugates. Through a detailed examination of the physicochemical properties and morphological features, this investigation sought to unravel the underlying molecular interactions between PDA NPs and the aptamer, providing insights crucial for the rational design and optimization of biosensors. With the goal of advancing the field of biosensor applications, this work contributes to the understanding of colloid chemistry of PDA NPs in the context of biohybrid materials between PDA NPs and DNA aptamers and opens possibilities for the development of a novel diagnostic tool for clinical use, as demonstrated by the sensitive detection of biomarkers such as glycated albumin (GA), which is a significant indicator for glycemic control in diabetes management. The concept of this study is shown in Scheme 1.

## 2 Experiments

### 2.1 Reagents and materials

An amine-modified aptamer with a 23-nucleotide sequence of 5′-NH_2_-TGC GGT TGT AGT ACT CGT GGC CG-3′ (Apiwat et al., 2016) was synthesized by Integrated DNA Technologies Pte. Ltd., Singapore. Dopamine hydrochloride (MW = 189.6), GA, and ethanolamine were purchased from Sigma‒Aldrich (Singapore). Disposable screen-printed carbon electrodes (SPCEs) were purchased from Quasense, Thailand. Potassium ferricyanide (K_3_ [Fe(CN)_6_]) at 5 mM with 0.1 M potassium chloride (KCl) in phosphate-buffered saline (PBS) (1X, pH 7.4) was used as a redox indicator. PBS was used for all washing steps. All reagents were prepared with ultrapure deionized water.

### 2.2 Instruments and apparatus

Electroanalysis was performed by cyclic voltammetry (CV), differential pulse voltammetry (DPV), and electrochemical impedance spectroscopy (EIS) methods using a PalmSens4 potentiostat instrument (PalmSens BV Co., Ltd., Netherlands) with the software PS trace 5.6. A system with three electrodes was utilized, which included a silver/silver chloride (Ag/AgCl) reference electrode, working (2.5 mm diameter) and counter electrodes made of carbon. The absorbance spectra of the PDA NPs were obtained with an Eppendorf BioSpectrometer^®^ fluorescence spectrophotometer (Hamburg, Germany). The size, structure, and surface morphology of the PDA NPs were measured by a Zetasizer Nano ZS instrument (Malvern, United Kingdom), a transmission electron microscopy (TEM, FEI, TECNAI G2 20, Nieuw-Vennep, Netherlands), and a scanning electron microscopy (SEM) (Jeol, JSM-IT200 InTouchScope™, Tokyo, Japan). Both the PDA NPs and PDA NP-aptamer conjugates were measured by a Bruker TENSOR II ATR-FTIR spectrometer (Bruker, Bremen, Germany) and an XploRA PLUS Raman spectrometer (Horbita, Kyoto, Japan).

### 2.3 Synthesis of polydopamine nanoparticles

PDA NPs were prepared by a simple process involving self-oxidative polymerization of dopamine in alkaline Tris buffer. Specifically, dopamine hydrochloride (0.025 g) was dissolved in 50 mL of 10 mM Tris buffer at pH 10.5. Using a magnetic stirrer, this solution was stirred mildly at room temperature for 20 h. The color of this solution immediately turned light brown and later became very dark brown. The resulting PDA NPs were separated by centrifugation at 16,100 *g* and rinsed two times with Tris buffer. This suspension was resuspended in 100 µL of Tris buffer for further experiments.

### 2.4 Molecular dynamics simulation

#### 2.4.1 General model preparation

**FIGURE 1:**
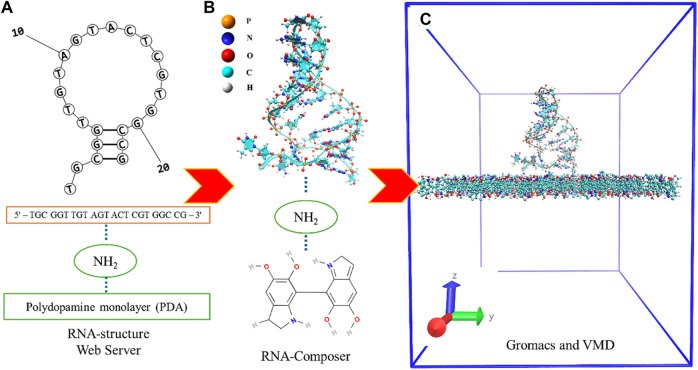
**(A)** Specific nucleotide sequence of the DNA aptamer of which the secondary structure was predicted and the proposed 2D structure of the DNA aptamer. **(B)** The 3D DNA-aptamer structure with an amino linker and a PDA monolayer. **(C)** The model system is in an initial simulation box of dimensions 7.85 × 7.85 × 10.0 nm^3^ without the aqueous solution.

The model preparation process involved three primary steps: 1) The initial configuration of the model system included PDA molecules, an NH_2_ linker, and a DNA aptamer, which was simply modeled in an aqueous solution via the procedural steps of other studies (Jeddi and Saiz, 2017; Oliveira et al., 2022; Lee et al., 2023). The DNA-aptamer secondary structure was predicted by determining the specific nucleotide sequence of the DNA aptamer (5′–TGC GGT TGT AGT ACT CGT GGC CG–3′). The RNA-structure webserver tool (Reuter and Mathews, 2010) was used to predict the secondary structure (2D) of the DNA aptamer by adding a nucleotide sequence, as shown in Figure 1A. 2) In the next step, as shown in Figure 1B, the 3D DNA-aptamer structure was generated using an RNA composer (Popenda et al., 2012). After obtaining the RNA structure, its structure was mutated from U to T by VMD (Humphrey et al., 1996), and the resulting structure was recorded as a protein data bank (PDB) file. 3) The initial configuration of the DNA-aptamer orientation with the NH_2_ linker attached to the PDA surface was determined by GROMACS (Abraham et al., 2015) and VMD (Humphrey et al., 1996), as shown in Figure 1C. The DNA aptamer attached to the NH_2_ linker had an initial orientation perpendicular to the PDA surface. The PDA structure (Chen and Buehler, 2018; Li et al., 2021) was created using the CELLmicrocosmos v.2.2 Membrane Editor software program (Sommer, 2019), which randomly arranged 175 molecules in a monolayer within a 7.85 nm × 7.85 nm box. Finally, the two primary groups of molecules were assembled into the system, and water molecules were added. An aqueous solution of extended simple point charge mode (SPC/E) was produced, which included 53,808 molecules of water. In the illustration, water molecules are not shown for clarity. All the molecular dynamics simulations were carried out using GROMACS (Abraham et al., 2015) with the topology file of the all-atom CHARMM force field for DNA aptamers (CHARMM27) (MacKerell et al., 2000), the ATB force field (Malde et al., 2011) for the NH_2_ linker and PDA layer, and the solvent SPC/E model for the aqueous solution.

#### 2.4.2 Simulation setup and parameters

The model system was first equilibrated at 200 ps with the NVT ensemble, and the energy was minimized using the steepest descent approach to remove the overlap between nearby atoms. Using the leapfrog technique for constant pressure and the NPT ensemble with a time step of 2 fs, an equilibration run of 1 ns was conducted. Using the LINCS method (Baranyai and Evans, 1990), all bond lengths and angles were calculated at a 2 fs integration time step. A grid spacing of 0.16 nm was used with a direct cutoff of 1.4 nm. The electrostatic interactions at long range were modeled using the particle mesh Ewald method (Darden et al., 1993). Using the Parrinello-Rahman barostat, a coupling time constant of 1.0 ps at 1 bar of system pressure was included (Parrinello and Rahman, 1981). Using the Nosé–Hoover thermostat method, the system temperature was controlled to 300 K with a coupling time constant of 0.5 ps (Evans and Holian, 1985). The MD production run was simulated at 30 ns. The MD trajectories were determined in GROMACS (Abraham et al., 2015), and Visual Molecular Dynamics (VMD) software (Humphrey et al., 1996) was used to generate all trajectories and graphical representations.

### 2.5 Electrochemical analysis

Electrochemical studies were performed on SPCEs that were attached to a PalmSens4 potentiostat through a connector. Seven microliters of bare PDA NP solution were deposited onto the surface of the working carbon electrode and incubated for 30 min. The electrode was washed with PBS to remove excess PDA NPs. After rinsing with PBS, 150 µL of 5 mM [Fe(CN)_6_]^4-/3-^ was added until all the electrode surfaces were immersed. EIS measurements were performed with a frequency range from 100 mHz to 100 kHz, and the AC potential was 0.025 V. The Randles circuit parameters were obtained by fitting the EIS data using the PSTrace software package. The charge transfer resistance (Rct) was calculated.

To fabricate PDA NP/aptamer/ethanolamine-modified SPCEs, equal volumes of PDA NP solution and different concentrations (0.05, 0.5, and 5 µM) of aptamer were conjugated at room temperature for 45 min. Seven microliters of PDA NP-aptamer conjugates were deposited onto the surface of the working carbon electrode and incubated for 30 min. The excess unbound conjugates were washed thoroughly with PBS (0.01 M, pH 7.4). Ethanolamine at a concentration of 0.1 M was used as a blocking reagent to reduce nonspecific binding on the electrode surface for 30 min, after which the electrode was washed with PBS. To characterize the fabrication process, CV and DPV analyses were carried out after each immobilization step. CV was carried out by applying a voltage ranging from −0.6 to 0.6 V, and the scan rate was set to 0.1 mVs^−1^. DPV analysis was performed using a voltage range from −0.015 to 0.6 V at a scan rate of 100 mVs^-1^. Different concentrations of GA (0.5, 1, 10, 100, and 1000 μg/mL) were incubated with the prepared aptasensor at three different aptamer concentrations (0.05, 0.5, and 5 µM) for 60 min. DVP analysis was performed in the same redox probe solution with a voltage ranging from −0.015 to 0.6 V at a scan rate of 100 mVs^−1^. The changes in the peak current were recorded to plot the calibration curve for GA concentrations.

### 2.6 Specificity of the developed aptasensor

The specificity of the PDA NP-functionalized aptasensor developed in this study for GA was evaluated by testing its response to various interfering substances commonly found in biological samples. Different concentrations of the aptamer specific to GA were used to assess the selectivity of the biosensor. A comparative analysis was conducted with human serum albumin (HSA), glucose, urea, bilirubin, folic acid, amoxicillin, uric acid, and vitamin C to investigate the ability of the biosensor to differentiate and selectively detect GA within a complex biological matrix.

### 2.7 Spike recovery assay

A recovery assay of the electrochemical aptasensor was performed by using GA-spiked human serum. First, human serum was diluted to 1:10,000 with 1× PBS at pH 7.4 to remove the other interferents. Two different concentrations of GA (52.5 and 262.5 μg/mL) were spiked into this diluted serum. Electrochemical determination and DPV analysis were performed according to the experimental procedure described in Section 2.5, Electrochemical Analysis. The % recovery rate was calculated by the formula below.

$$\%\text{Recovery}=\frac{\left(\text{spiked}\;\text{sample}\;\text{result}-\text{unspiked}\;\text{sample}\;\text{result}\right)}{\text{Known}\;\text{add}\;\text{spiked}\;\text{concentration}}\times 100$$

## 3 Results and discussion

### 3.1 PDA synthesis and characterization

**FIGURE 2:**
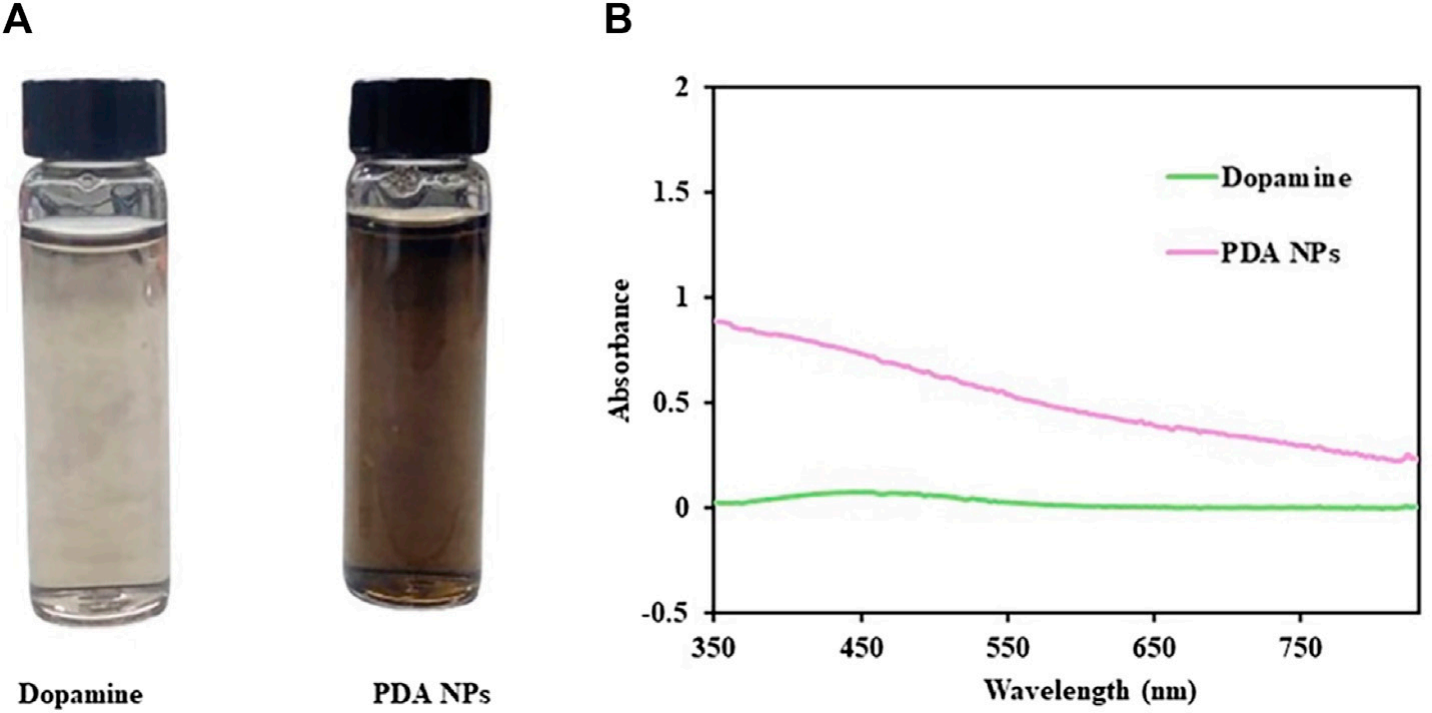
**(A)** The solution of dopamine and PDA NPs. **(B)** UV‒vis absorption spectra of dopamine and PDA NPs.

The newly synthesized PDA NPs were obtained through the oxidative polymerization of dopamine dissolved in Tris buffer at pH 10.5. The appearance of the PDA NPs changed gradually from light brown to dark brown, as observed by the naked eye (Figure 2A). This finding indicated that it is a successful synthesis of PDA NPs (Ball, 2018). The UV–vis absorption spectra of the synthesized PDA NPs and DA solution at wavelengths ranging from 350 to 830 nm are shown in Figure 2B. The DA monomer showed no absorption, while PDA NPs exhibited a pattern of decreasing absorbance with increasing wavelength.

**FIGURE 3:**
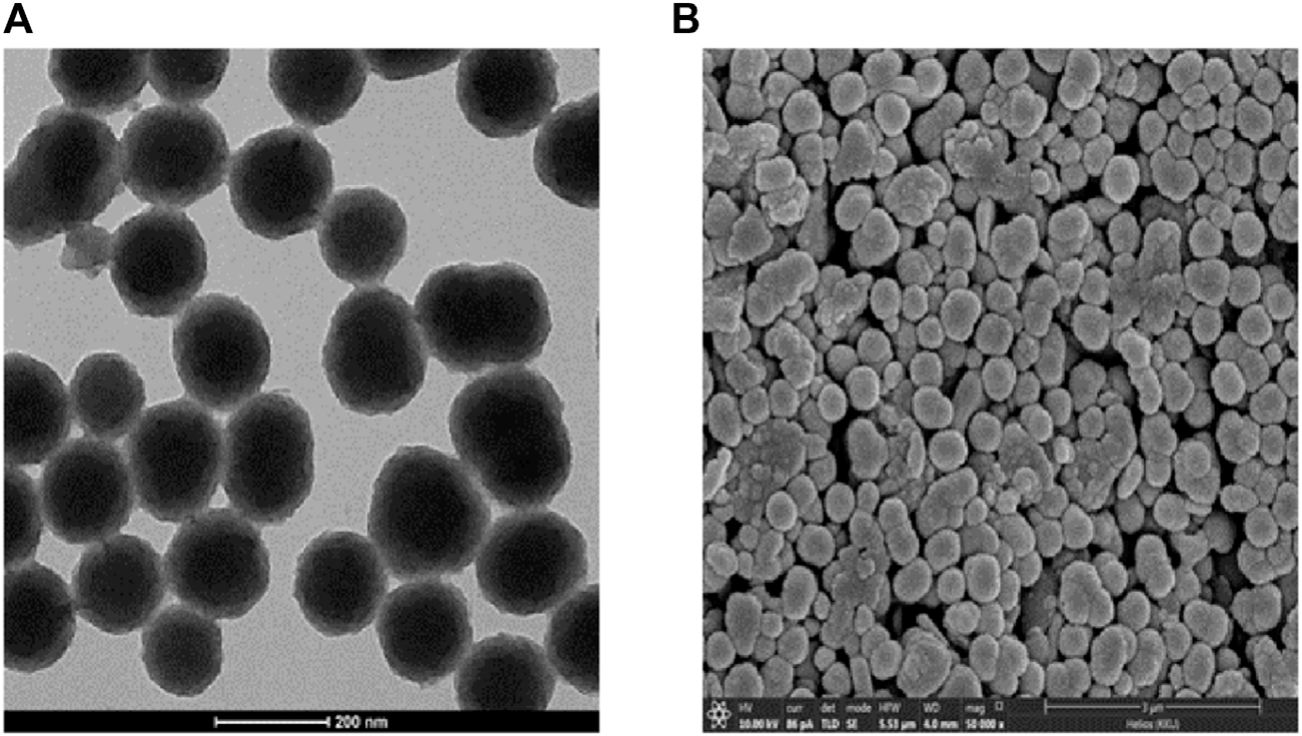
**(A)** TEM and **(B)** SEM images of PDA NPs.

PDA NPs were dropped onto carbon-coated copper grids and SPCEs to facilitate their examination under TEM and SEM, respectively. The TEM and SEM images in Figures 3A,B show the morphology of the PDA NPs. The PDA NPs exhibited a spherical shape and a consistent size distribution on the substrates.

### 3.2 Conjugation of anti-GA aptamers to the surfaces of PDA NPs

PDA NPs express important functional groups (e.g., catechol, amine, and imine) that can be used to covalently immobilize molecules (Jia et al., 2019). Therefore, amine-modified aptamers were introduced to functionalize PDA NPs via Michael addition and/or Schiff base reactions (Zhong et al., 2015). DLS measurements were conducted to assess the particle size and polydispersity index (PDI) of the PDA NPs, both in their bare state and following conjugation with the aptamers at varying concentrations. The results (summarized in Table 1) demonstrated a substantial impact of aptamer conjugation on the particle size and distribution. The initial analysis revealed that the bare PDA NPs had an average hydrodynamic diameter of 155.3 ± 1.32 nm and a narrow PDI, suggesting a remarkably uniform population. The subsequent conjugation with the aptamers led to an increase in the particle size, which varied depending on the concentration. These findings demonstrated the effective modification of the physicochemical properties of the PDA NPs upon aptamer conjugation, leading to the formation of larger and more heterogeneous particles. Such changes in particle size and distribution can significantly impact the performance and application of the resulting biosensor, highlighting the importance of precise control and characterization of the conjugation process in the design of biosensing platforms.

**TABLE 1:** DLS measurements of unmodified and modified PDA NPs.

Sample	Particle size (nm)	PDI
Bare PDA NPs	155.3 ± 1.32	0.01 ± 0.005
PDA NPs + 0.05 µM aptamer	226.7 ± 3.66	0.28 ± 0.035
PDA NPs + 0.5 µM aptamer	333.8 ± 10.54	0.42 ± 0.029
PDA NPs + 5 µM aptamer	411.2 ± 38.70	0.50 ± 0.045

**FIGURE 4:**
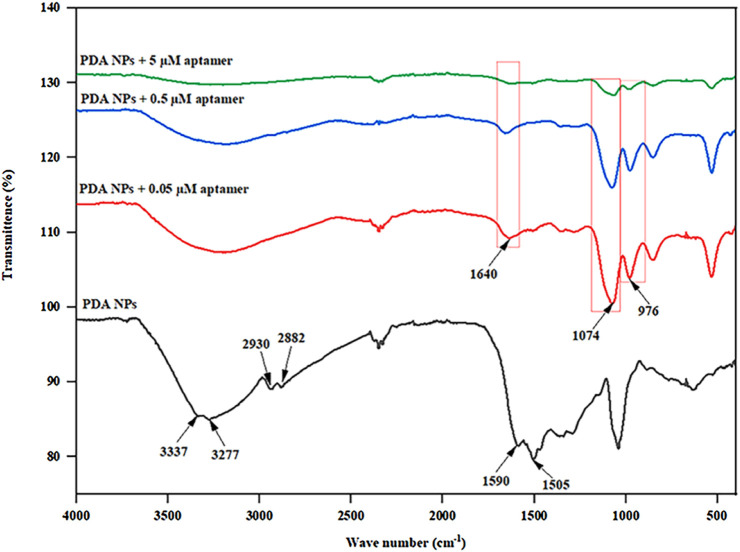
FTIR spectra of the PDA NPs and PDA NPs conjugated with different aptamer concentrations (0.05, 0.5, and 5 µM).

FTIR analysis, as shown in Figure 4, was conducted to elucidate the functional groups and molecular interactions present in the bare PDA NPs and the PDA NPs conjugated with different concentrations of the aptamers (0.05, 0.5, and 5 µM). The FTIR spectra revealed distinct peak patterns corresponding to various functional groups within the samples. The peaks observed in the FTIR spectra were indicative of specific molecular vibrations within the samples. For instance, the peaks at approximately 3337 cm^−1^ and 3277 cm^−1^ are attributed to N-H and O-H stretching vibrations (Luo et al., 2015; Huang et al., 2018; Thakur et al., 2018), respectively, while the peaks at approximately 2930 cm^−1^ and 2882 cm^−1^ are associated with C-H stretching vibrations from the aromatic ring of the PDA NPs (Cui et al., 2016; Moraes et al., 2016; Yang et al., 2017). The peaks at approximately 1590 cm^−1^ and 1505 cm^−1^ are associated with C=C and C-N stretching vibrations from the indole group of the PDA NPs (Wang et al., 2002; Batul et al., 2018; Bhardwaj et al., 2020; Narayanan et al., 2023), respectively. The emergence of a peak at 1640 cm^⁻1^ in conjugated samples corresponds to the amide I band, which typically arises from C=O stretching vibrations in peptide bonds (Delgado-López et al., 2012; Sadat and Joye, 2020). Additionally, the peak at approximately 1074 cm^−1^ corresponds to the P-O stretching vibration of phosphates in nucleotides (Delgado-López et al., 2012; Li et al., 2016; Iconaru et al., 2022; Duan et al., 2023), while the peak at approximately 976 cm^−1^ is attributed to C-C/C-O stretching vibrations from the characteristic sugar-phosphate backbone found in DNA (Tsuboi, 1970; Mello and Vidal, 2012; Ranu et al., 2019), supporting the successful conjugation of the aptamer on the surface of PDA NPs. The relative intensities of these peaks provide crucial insights into the compositional changes induced by aptamer conjugation. For instance, the weaker intensity at 3337 cm^−1^ in the spectra of the aptamer-conjugated PDA NPs compared to that of the bare PDA NPs indicated a reduction in N-H stretching. This suggests that the binding of the aptamers to the PDA surface may have obstructed the N-H groups, potentially altering their vibrational behavior. The FTIR study thus provides valuable evidence on the molecular-level interactions between the PDA NPs and the aptamers, shedding light on the surface functionalization induced by the conjugation process. These findings further support the rationale for the observed alterations in physiochemical properties and the potential to influence biosensor performance through the creation of a bioactive interface capable of specific molecular recognition.

### 3.3 Raman spectroscopy

**FIGURE 5:**
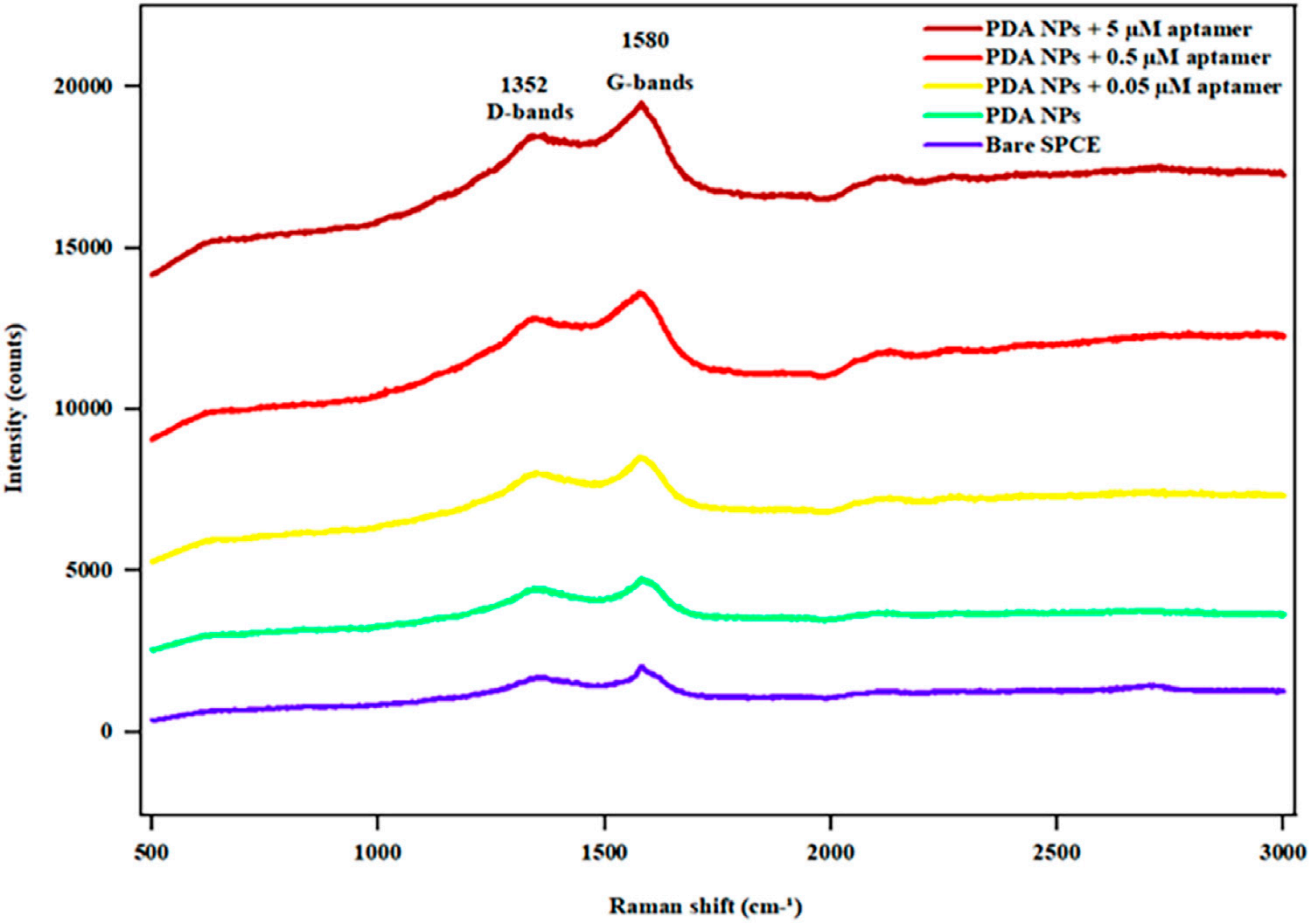
Raman spectra of the bare SPCE, PDA NPs, and aptamer-functionalized PDA NPs at different aptamer concentrations (0.05, 0.5, and 5 µM).

Raman spectroscopy was performed to investigate the structure of the PDA NPs and PDA NP-aptamer conjugates. As shown in Figure 5, two characteristic peaks were observed in the Raman spectra, corresponding to the D band at 1352 cm^−1^ and the G band at 1580 cm^−1^ (Ranu et al., 2019). The D band is attributed to the sp2 carbon of a disordered, defect-rich, or amorphous carbon structure, while the G band corresponds to the sp2 carbon in the graphitic lattice. In this study, the intensity ratio of the D and G bands (I_D_/I_G_) for the PDA sample was 0.88, while the value of I_D_/I_G_ was slightly reduced to 0.87, 0.85, and 0.84 for PDA NP-aptamer conjugates at 0.05, 0.5, and 5 μM, respectively, suggesting that the conjugates slightly increased the graphitic phases with a high concentration of aptamer (Yang et al., 2020), indicating that distinct Raman signal changes were observed only upon binding of the target molecule.

### 3.4 Molecular dynamics simulation

**FIGURE 6:**
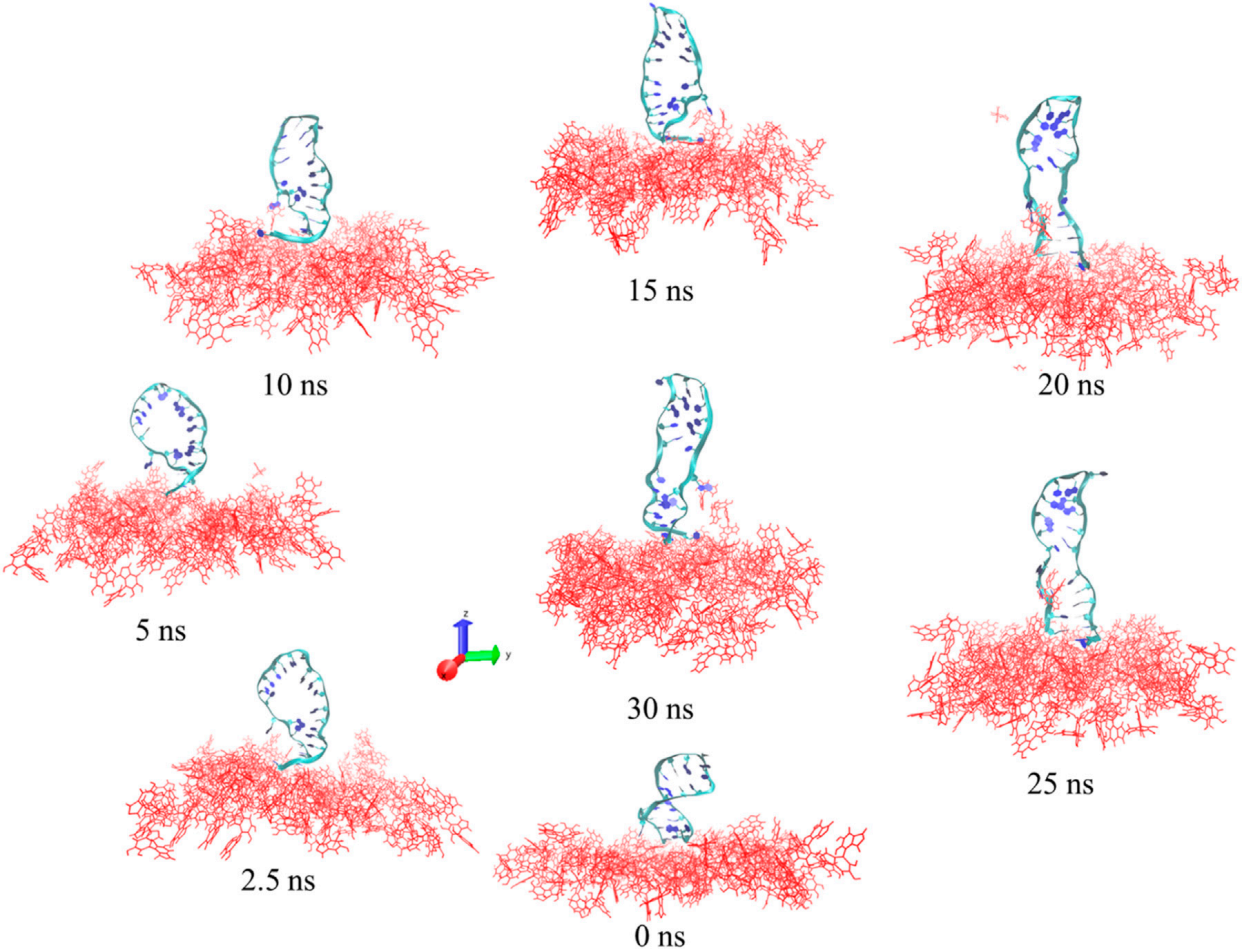
Images of the DNA aptamer with the NH_2_ linker attached to the PDA layer at 0 ns, 2.5 ns, 5 ns, 10 ns, 15 ns, 20 ns, 25 ns, and 30 ns. The DNA-aptamer residues are shown as ribbons, and the PDA layers are displayed in red.

Screenshots from the simulation for a total of 30 ns of the NH_2_ linker linked to the PDA surface by the DNA aptamer are displayed in Figure 6. As demonstrated in Figure 6, the DNA aptamer tended to lean or curl over time before gradually ceasing to move (20 ns–30 ns). The area per molecule on the PDA surface also tended to decrease over time. The DNA aptamers displayed twisting and unscrewing ribbons early in the simulation. The stretching and relaxation of the DNA aptamer structures over 15–30 ns of simulation time on a screw ribbon revealed that the PDA surface had stabilized into an upright, perpendicular orientation after 20 ns and continued to do so for the next 30 ns. The backbone DNA aptamer structure was stretched in this orientation due to structural flexibility or interactions with nearby water molecules. The stretching of the DNA aptamer structure functionalized on the PDA surface was found to be consistent with experimental observations, which showed notable changes in particle size, FTIR, and Raman spectroscopy measurements, as mentioned above. The computational models elucidate the formation of DNA aptamer with NH_2_ linker on the PDA surface, illustrating how this interaction directly influences the conformational size of the whole system, as evidenced by the experimental observations. Despite performing the simple models, the parameters such as room temperature and aqueous solvent conditions were aligned with those of the experimental settings. Consequently, the simulation results corroborate the structural changes in the DNA aptamer and the NH_2_ linker upon integration into the system. This not only impacts their conformation but also enhances the comprehension of DNA-aptamer interactions with the PDA surface. However, previous experimental and computational studies have demonstrated that the orientation or conformation of a tethered biorecognition element on a biosensor surface can alter upon immobilization, significantly affecting the biosensor’s performance (Wong and Pettitt, 2004; Jeddi and Saiz, 2021).

### 3.5 Electrochemical analysis

#### 3.5.1 Characterization of the PDA NPs

**FIGURE 7:**
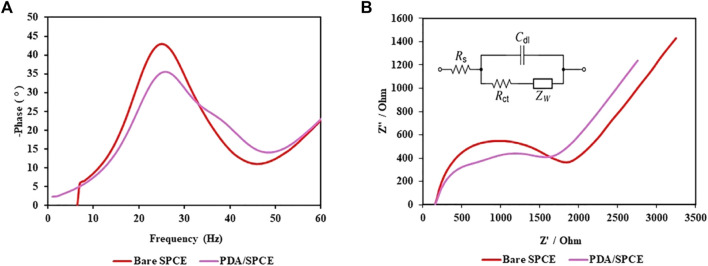
EIS spectra of the bare SPCE and PDA/SPCE: **(A)** Bode plot and **(B)** Nyquist plot.

Figure 7A shows the Bode plot for bare and PDA NP-modified SPCEs indicating distinct electrochemical behaviors. The PDA NP-modified SPCE consistently showed lower phase shift angles. Figure 7B shows the Nyquist plots of the bare SPCE and PDA/SPCE in a 1 M KCl solution containing 5 mM [Fe(CN)_6_]^4-/3-^. EIS analysis involves plotting so-called Nyquist plots showing the real and imaginary parts of the complex impedance of individual electrodes or electrochemical cells (Mei et al., 2018). It is a useful tool for studying the charge transfer properties of electrodes. The diameter of the semicircle in the Nyquist plot is related to the charge transfer resistance (Rct) of the electrode. The larger the diameter is, the greater the Rct (Magar et al., 2021). Figure 7B shows that the diameter of the semicircle for the PDA/SPCE was smaller than that for the bare SPCE. This indicates that the Rct of the PDA/SPCE electrode was lower than that of the bare SPCE electrode. In other words, the PDA modification improved the charge transfer properties and surface area of the SPCE electrode, which can lead to higher current densities. This is likely because a unique PDA composite can be used as a conductive polymer, which could lead to the development of more sensitive electrochemical sensors, biosensors, bioelectronics, and bionic interfaces (Eom et al., 2022).

#### 3.5.2 Characterization of the polydopamine nanoparticles-functionalized aptasensor

PDA NPs were utilized as surface-active agents to facilitate the conjugation of DNA aptamers directly onto carbon electrodes without the need for additional chemical treatments. Previous studies in this field have explored thiol- or amine-terminated polymers grafted onto PDA-coated surfaces through thiol-catechol or amine-catechol adducts via Michael-type addition reactions and Schiff-base formation (Ryu et al., 2018; Manolakis and Azhar, 2020). These methodologies have been extended to incorporate various biomolecules, such as peptides, protein enzymes, and oligonucleotides. The direct conjugation of PDA NPs and aptamers offers a simplified and efficient approach for surface functionalization without the need for additional coupling reagents such as EDC-NHS. In this experiment, equal volumes of PDA NPs and aptamers were directly conjugated by covalent bonding through either Michael-type addition reactions or Schiff base formation. The conjugates had stable and efficient attachment without the complications associated with chemical cross-linking.

**FIGURE 8:**
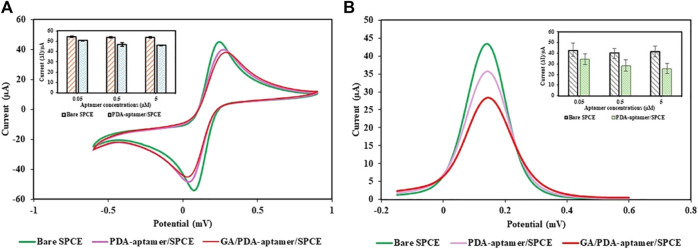
Electrochemical study of the proposed electrochemical PDA NP-functionalized aptasensor: **(A)** CV and **(B)** DPV.

The constructed PDA NP-modified electrochemical aptasensor was characterized by CV and EIS using 5 mM [Fe(CN)_6_]^3−/4−^. Figure 8A shows the CV curves of the different electrodes. When the bare SPCE was immobilized with the PDA NP-aptamer conjugates, the peak current of the redox probe slightly decreased, accompanied by a slight increase in the peak-to-peak potential difference. After the nonspecific sites on the electrode were blocked using ethanolamine, the redox current slightly decreased owing to the nonconductive nature of the immobilized aptamer and the blocked ethanolamine. After being incubated with target molecules, the electrochemical biosensor had a small peak current and a large peak-to-peak potential difference. The CV data demonstrated the effective preparation of the electrochemical biosensor. The DPV spectra of the electrochemical biosensor are shown in Figure 8B. The DPV curve for the PDA NP-aptamer-modified SPCE shows a decrease in the peak current compared to that of the bare SPCE. This is because the PDA NP-aptamer coating hindered electron transfer between the redox probe and the electrode surface. The DPV curve for the GA/PDA NP-aptamer-modified SPCE showed a further decrease in the peak current compared to that of the PDA NP-aptamer-modified SPCE. This is because the target molecules were bound to the conjugates on the electrode surface, further hindering electron transfer. The CV and DPV data suggested that the electrochemical biosensor successfully immobilized each layer and successfully detected GA.

#### 3.5.3 Analytical performance of the PDA NP-modified electrochemical aptasensor

**FIGURE 9:**
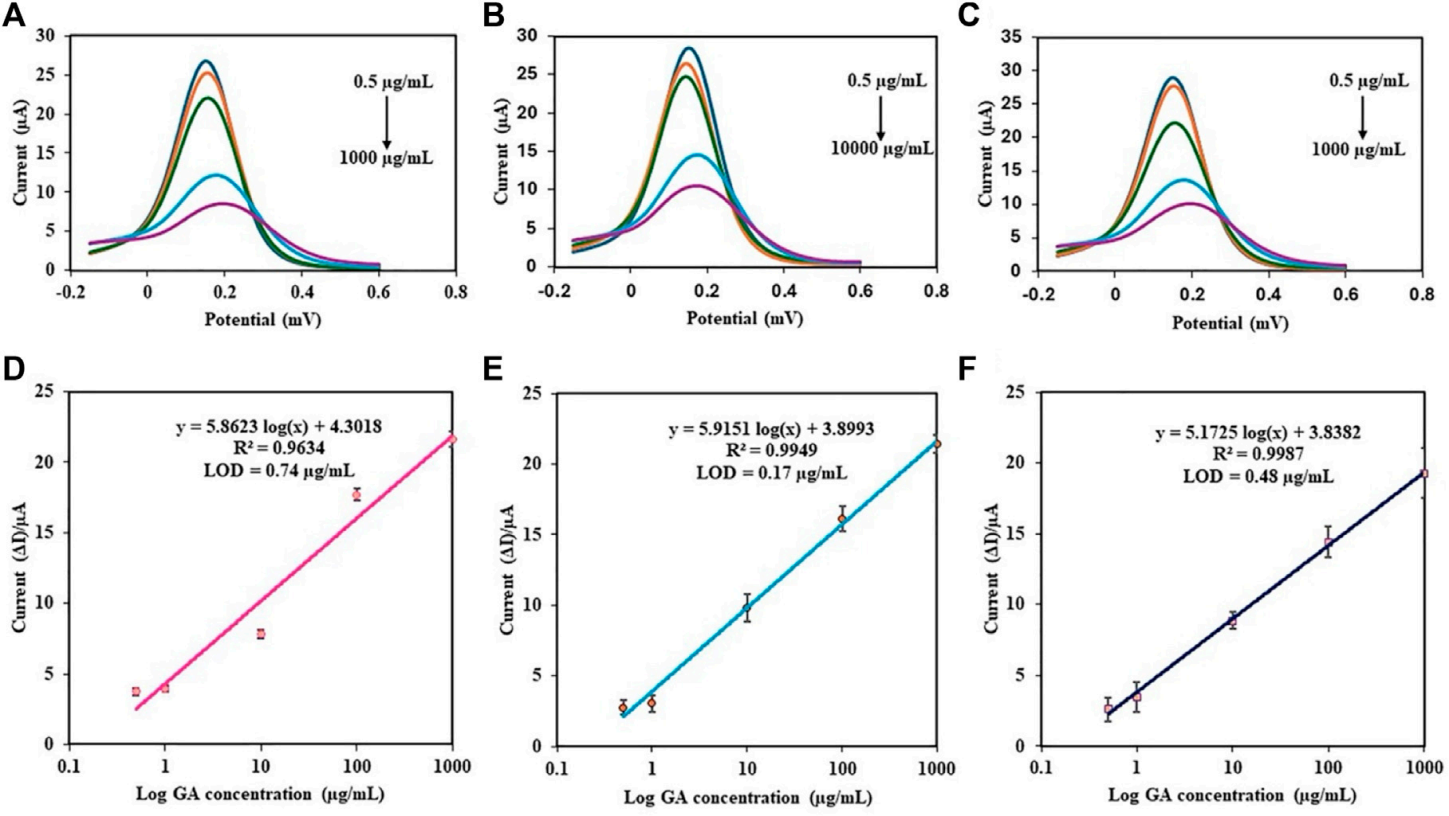
DPV responses of the different concentrations of GA in PBS using the PDA NP-modified electrochemical aptasensor at various aptamer concentrations: **(A)** 0.05 µM, **(B)** 0.5 µM, and **(C)** 5 µM. Calibration plots of the aptasensor using different aptamer concentrations: **(D)** 0.05 µM, **(E)** 0.5 µM, and **(F)** 5 µM.

The analytical performance of the PDA NP-modified electrochemical aptasensor was evaluated by conducting DPV measurements over a wide range of GA concentrations (0.5–1000 μg/mL). The DPV peak current was observed to decrease with increasing concentration of GA (Figures 9A–C). As shown in Figures 9D–F, the results showed that the aptasensor performance varied with the concentration of aptamer used. The coefficient of determination (*R* ^2^) is a statistical measure that represents the proportion of the variance for a dependent variable that is explained by an independent variable or variables in a regression model. An *R* ^2^ of 0.9634 at 0.05 µM, 0.9949 at 0.5 µM, and 0.9987 at 5 µM indicated that the aptamer concentration significantly improved the aptasensor’s predictive ability, suggesting that the aptasensor is highly sensitive to changes in the aptamer concentration. The limit of detection (LOD) is the lowest concentration of an analyte that can be reliably detected by a method. A lower LOD indicates a greater sensitivity of the aptasensor. The LOD of GA was calculated via the following equation: LOD = 3σ/S, where σ is the standard deviation of the blank solutions and S is the slope of the calibration curve (Yang et al., 2015). The LODs were 0.74, 0.17, and 0.48 μg/mL as the aptamer concentrations of 0.05, 0.5, and 5 μM, respectively. This indicates that the sensitivity of the aptasensor improved when the optimal aptamer concentration was 0.5 µM, as this concentration provided the best balance between linearity and sensitivity. Although the aptamer concentration of 5 µM seems more promising since it has the highest *R* ^2^ for the GA standard curve compared to the other two aptamer concentrations, the calculated LOD of GA was the best at an aptamer concentration of 0.5 µM. Hence, the proposed aptasensor at an aptamer concentration of 0.5 µM exhibited excellent activity toward the determination of GA.

Table 2 shows the aptamer sequence, LOD, and linear range of various other aptamer-based biosensors for GA detection compared to our study.

**TABLE 2:** Comparison of aptamer sequence, LOD, and linearity from various aptamer-based biosensors for GA detection.

Method	Aptamer sequence	LOD	Linear sensing range	Ref.
Optical aptasensor	5′Amino C6/TGCGGTTGTAGTACTCGTGGCCG/Thiol C6 SS 3′	0.067 μg/mL	0–900 μg/mL	Ghosh et al. (2017)
Reduced graphene/Au-based electrochemical aptasensor	5′GGT​GGC​TGG​AGG​GGG​CGC​GAA​CGT​TTT​TTT​TTT 3′– SH	0.07 μg/mL	2–10 μg/mL	Farzadfard et al. (2020)
Fluorescent quenching of graphene oxide and Cy5-labeled G8 aptamer	5′GGT​GCG​GTT​CGT​GCG​GTT​GTA​GTA​CTC​GTG​GCC​GAT​AGA​GGT​AGT​TTC​G 3′	50 μg/mL	50–300 μg/mL	Apiwat et al. (2016)
Fluorescence quenching of reduced graphene oxide and FAM-labeled aptamer	5′ATG​CGG​ATC​CCG​CGC​GCA​GTG​CAG​GGA​GCC​GCT​CCA​CGT​ACG​TTG​CGC​GAA​GCT​TGC​GC 3′	16.40 μg/mL	0–125 μg/mL	Kim et al. (2020)
PDA NPs-functionalized electrochemical aptasensor at 0.5 µM aptamer concentration	5′-NH_2_-TGCGGTTGTAGTACTCGT GGCCG 3′	0.17 μg/mL	0.5–1000 μg/mL	This work

#### 3.5.4 Interference study

Selectivity testing (Supplementary Figure S1) using DPV revealed good discrimination of GA (1 mg/mL) from potential interferents such as HSA (100 μg/mL), glucose (125 mg/dL), bilirubin (2 mg/dL), urea (2.5 mg/mL), folic acid (160 μg/mL), amoxicillin (5 mg/mL), vitamin C (5 mg/mL), and uric acid (3 mg/mL) at all aptamer concentrations (0.05, 0.5, and 5 µM). There were significant differences in the selectivity of the aptasensor among the different aptamer concentrations. This suggests the best selectivity at 0.5 µM aptamer concentration toward GA in the presence of these common biological interferents.

#### 3.5.5 Spike recovery assay

PDA NPs-functionalized aptasensors at three different concentrations (0.05, 0.5, and 5 µM) were tested in a recovery assay using two target concentrations of GA spiked in serum (52.5 and 262.5 μg/mL) (Table 3). The measured concentrations of GA in serum after aptasensor analysis were compared to the spiked concentrations to determine the recovery percentage and relative standard deviation (RSD). The recovery percentages for all six conditions ranged from 94.4% to 122.4%, indicating that the aptasensor can effectively capture the target molecule at all three concentrations and for both target concentrations. The lowest recovery was observed at a 0.05 μM aptasensor concentration of 262.5 μg/mL, which could be due to saturation of the binding sites at higher target concentrations. The highest recovery was observed at a 5 μM aptasensor concentration with a 52.5 μg/mL target concentration, which suggests that this concentration of the aptasensor may be most efficient for capturing the target molecule at lower concentrations. The RSD values were all below 14%, indicating good reproducibility of the assay. Therefore, the results suggested that the 0.5 µM aptasensor performed the best in terms of both accuracy and precision for the detection of GA in serum, providing reliable measurements across a range of spiked concentrations.

**TABLE 3:** Recovery assay of GA protein in human serum using the developed aptasensor.

Aptasensor concentration (µM)	GA concentration spiked in serum (µg/mL)	Measured concentration (µg/mL)	Recovery (%)	RSD (%) (n = 3)
0.05	52.5	54.2 ± 3.6	103.3	6.7
	262.5	248.0 ± 34.4	94.4	13.9
0.5	52.5	51.4 ± 0.4	97.9	0.8
	262.5	266.5 ± 2.6	101.5	0.9
5	52.5	64.3 ± 4.9	122.4	7.6
	262.5	307.2 ± 18.2	117.0	5.9

## 4 Conclusion

The synthesis and functionalization of PDA NPs with DNA aptamers for biosensing applications were thoroughly investigated in this study. The findings demonstrated the significant impact of DNA aptamer concentration on the properties and functionalities of PDA NPs, with notable changes in particle size, FTIR, and Raman spectroscopy measurements. We first reported MD simulation studies of DNA aptamer to GA and PDA NPs. The equilibrated structure of the DNA aptamer with the NH_2_ linker attached to the PDA surface showed a hairpin, stretching, and orientation, including stabilization along the perpendicular axis with the PDA surface. Changes in conformation can affect biosensor properties. The electrochemical properties of the PDA NPs and PDA NPs functionalized aptasensor were also thoroughly examined, revealing that the optimal PDA NPs coated SPCEs decreased the charge transfer resistance, and the PDA NPs functionalized aptasensor demonstrated the potential for high sensitivity in the detection of GA in serum samples. The optimal aptamer concentration for achieving the best performance of the aptasensor was 0.5 µM, indicating that the aptasensor’s sensitivity can be precisely controlled by adjusting the aptamer concentration. These results underscore the potential of PDA NPs as a versatile platform for the development of sensitive and specific biosensors for various applications, including clinical diagnostics and environmental monitoring. Future studies should include the validation of real samples, comparative analyses with existing biosensing platforms, and investigations of long-term stability. Addressing these limitations would enhance the understanding of aptasensor behavior and facilitate their practical deployment in biomedical and clinical settings.

## Data Availability

The original contributions presented in the study are included in the article/Supplementary Material, further inquiries can be directed to the corresponding author.
