# Supplementary material for: DNA aptamer-functionalized PDA nanoparticles: from colloidal chemistry to biosensor applications
Source: Front Bioeng Biotechnol. 2024 Jul 9;12:1427229. doi: 10.3389/fbioe.2024.1427229 (PMC11263086; doi:10.3389/fbioe.2024.1427229)
Supplement: Supplementary file 1 [file Image1.pdf]

Supplementary Material

Figure S1:

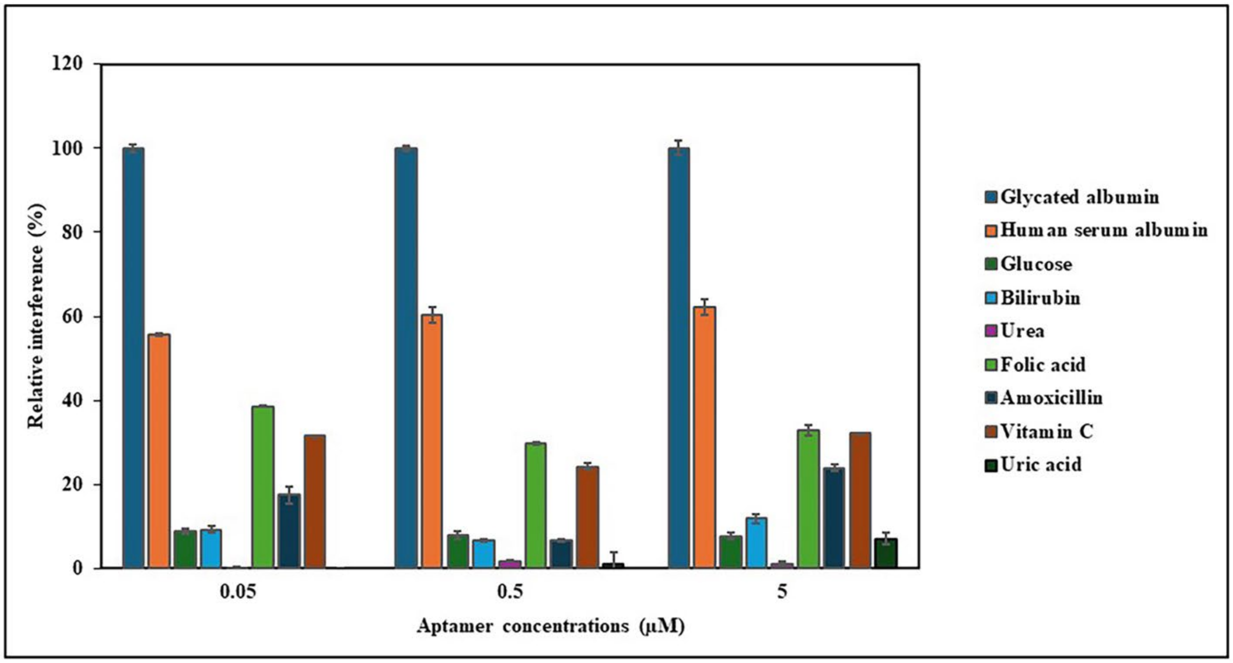

Figure S1. Interference study: the relative interference (%) of the proposed aptasensor at different aptamer concentrations for GA (1 mg/mL), human serum albumin (100 µg/mL), glucose (125 mg/dL), bilirubin (2 mg/dL), urea (2.5 mg/mL), folic acid (160 µg/mL), amoxicillin (5 mg/mL), vitamin C (5 mg/mL), and uric acid (3 mg/mL).
